# Dysbiosis of gut microbiota during fecal stream diversion in patients with colorectal cancer

**DOI:** 10.1186/s13099-023-00566-9

**Published:** 2023-08-18

**Authors:** Soo Young Lee, Hyeung-Min Park, Chang Hyun Kim, Hyeong Rok Kim

**Affiliations:** Department of Surgery, Chonnam National University Hwasun Hospital and Medical School, 322 Seoyang-Ro Hwasun-Eup, Hwasun-Gun, Jeonnam 58128 South Korea

**Keywords:** Fecal stream diversion, Microbiota, Colorectal cancer, Ileostomy, Dysbiosis

## Abstract

**Background:**

The effect of fecal stream diversion on the gut microbiota is still uncertain. The present study was designed to assess the effect of fecal stream diversion on the composition of the gut microbiota in patients with colorectal cancer. We included patients undergoing left-sided colorectal cancer surgery with (ileostomy group) or without (control group) diverting ileostomy. Fecal samples were collected from 10 patients in each group before surgery (*t*_*1*_) and after ileostomy repair in the ileostomy group and 6–12 months after the initial surgery in the control group (*t*_*2*_). The fecal microbiota was assessed using 16S rRNA sequencing, and changes in the composition of the fecal microbiota were compared between the two groups.

**Results:**

Alpha diversity analysis revealed that the complexity of fecal microbiota decreased between *t*_*1*_ and *t*_*2*_ only in the ileostomy group. Beta diversity analysis also showed dissimilarity between *t*_*1*_ and *t*_*2*_ only in the ileostomy group. The composition of the microbiota was similar between the two groups at *t*_*1*_. However, at *t*_*2*_, the ileostomy group had lower proportion of beneficial bacteria (Lachnospiraceae, 3.8% vs. 29.9%, *p* < 0.001; Ruminococcaceae, 0.6% vs. 18.4%, *p* < 0.001; *Blautia*, 0.1% vs. 9.1%, *p* < 0.001; *Faecalibacterium*, 0.2% vs. 7.5%, *p* < 0.001) and a higher proportion of harmful bacteria (Proteobacteria, 17.9% vs. 5.1%, *p* = 0.006; *Clostridium*, 16.2% vs. 1.1%, *p* = 0.013; *Streptococcus*, 17.7% vs. 1.6%, *p* = 0.002) than the control group.

**Conclusions:**

Fecal stream diversion was closely associated with less diversity and dysbiosis of the gut microbiota.

**Supplementary Information:**

The online version contains supplementary material available at 10.1186/s13099-023-00566-9.

## Background

The human intestine is inhabited by the gut microbiota, a vast assemblage of microorganisms including bacteria, fungi, archaea, viruses, and protozoa, which plays a significant role in maintaining human health [1, 2]. Sustaining a symbiotic association with the intestinal mucosa, the gut microbiota provides significant immunological, metabolic, and gut-protective functions in healthy individuals [2]. A depleted microbial biodiversity within the gut microbiota may increase the risk of developing various diseases [1]; therefore, understanding and preserving the delicate balance of the gut microbiota is critical for promoting human health. There are various factors that can affect the gut microbiota, such as the method of delivery and feeding, lifecycle stage, composition of diet, geographical location, pharmaceutical usage, and physiological and psychological stress [1]. Among these factors, the gut microbiota is highly responsive to diet, and a varied and complex diet is linked to a more diversified microbiota. The consumption of dietary fiber from fruits, vegetables, and other plant sources is associated with significant and meaningful changes in the gut microbiota, highlighting the potential for dietary alterations to impact intestinal health [1]. To gain a comprehensive understanding of intestinal health maintenance, it is important to investigate the impact of prolonged fasting on the gut microbiota. This can provide valuable insights into how dietary habits and other lifestyle factors influence gut microbial communities and overall intestinal health.

Although intermittent fasting can have a positive effect on the composition of the gut microbiota and result in improvement of insulin sensitivity and weight control [3], a longer fasting period may yield completely different results from intermittent fasting. There have been several studies regarding the impact of starvation on the gut microbiota in humans and other vertebrates [4–7]. Some animal studies have reported that hibernating animals had a decrease in microbial richness and diversity [4, 5]. However, it is difficult to investigate the effect of long-term fasting on the gut microbiota in humans. Patients with anorexia nervosa have been reported to have different compositions of the gut microbiota compared with normal-weight individuals [5]; in particular, the alpha diversity was lower in patients with anorexia nervosa [6, 7]. However, it is challenging to attribute the changes in the gut microbiota entirely to prolonged fasting in individuals with eating disorders because their psychopathological condition may also play a role. Therefore, comparison of the gut microbiota composition in patients with colorectal cancer with and without a diverting ileostomy will enable the identification of significant alterations in the gut microbiota during several months of bowel rest.

Diverting ileostomy is often constructed to lower the occurrence and clinical severity of anastomotic leak in colorectal cancer surgery [8]. Usually, diverting ileostomy is temporary, and a second operation for ileostomy closure is performed a few months after the initial surgery. However, during the maintenance of diverting ileostomy for several months, fecal stream diversion can cause inflammation in the defunctioned colon, which is called diversion colitis. Potential pathogenic factors of diversion colitis include a shortage of nutrients produced from anaerobic bacterial fermentation, such as short-chain fatty acids, a deficiency of oxidative substrates, alterations in the colonic mucosa, and the presence of harmful bacteria [9, 10]. From this perspective, it can be inferred that fecal stream diversion may disrupt the homeostasis of gut microbiota and reduce its diversity.

There have been a few studies about the relationship between fecal diversion and the gut microbiota [8, 11–13]. Williams and colleagues indicated that there was a reduction in the circular muscle contraction and smooth muscle area of the distal limb of the loop ileostomy, which could potentially cause decreased intestinal function [8]. Some other studies have also reported weakened intestinal barrier function, an altered intestinal environment [11, 12], and decreased diversity of the mucosa-associated microbiota [11, 13] in the defunctioned ileum. However, despite the significant impact that fecal diversion can have on the gut microbiota, there is a paucity of research investigating this phenomenon through fecal testing.

Therefore, the primary objective of this study was to investigate the impact of fecal stream diversion on gut microbiota composition and diversity using fecal testing as a key investigative tool. By doing so, we aimed to establish a theoretical basis for managing patients with diverting ileostomy in colorectal cancer surgery.

## Results

### Baseline characteristics

Table 1 shows the baseline characteristics of the enrolled patients. Clinical factors such as sex (*p* = 0.628), age (*p* = 0.174), body mass index (*p* = 0.757), and American Society of Anesthesiologists score (*p* = 0.628) were similar between the two groups. However, patients in the ileostomy group had upper (40.0%) and mid-to-low (60.0%) rectal cancers, whereas those in the control group had left-sided colon cancers (60.0%, *p* = 0.002). Accordingly, the surgical method was also different between the two groups (*p* = 0.007). Although the clinical (*p* = 0.513) and pathologic (*p* = 0.753) tumor stages were similar between the two groups, the ileostomy group had a higher proportion of patients who underwent nCRT than the control group (80.0% vs. 0.0%, *p* = 0.001), and the patients in the ileostomy group tended to have received more adjuvant chemotherapy (100.0% vs. 60.0%, *p* = 0.087) than those in the control group. The time interval between *t*_*1*_ and *t*_*2*_ was shorter in the ileostomy group than in the control group (6.0 ± 1.9 vs. 8.0 ± 2.1 months, *p* = 0.038).

**Table 1:** Baseline characteristics

	Ileostomy (n = 10)	Control (n = 10)	*p*
Sex			
Male	8 (80)	6 (60)	0.628
Female	2 (20)	4 (40)	
Age (years)	57.6 ± 7.8	63.1 ± 9.5	0.174
BMI (kg/m^2^)	24.1 ± 2.9	23.7 ± 3.6	0.757
ASA score			
2	8 (80)	6 (60)	0.628
3	2 (20)	4 (40)	
Location			
Sigmoid and rectosigmoid colon	0 (0)	6 (60)	0.002
Upper rectum	4 (40)	4 (40)	
Mid-to-low rectum	6 (60)	0 (0)	
Surgery			
AR	0 (0)	6 (60)	0.007
LAR	7 (70)	4 (40)	
uLAR (hand-sewn)	3 (30)	0 (0)	
Clinical TNM stage			
I	1 (10)	3 (30)	0.513
II	2 (20)	2 (20)	
III	7 (70)	5 (50)	
Pathologic TNM stage			
0 (complete response)	1 (10)	0 (0)	0.753
I	2 (20)	3 (30)	
II	4 (40)	4 (40)	
III	3 (30)	3 (30)	
Neoadjuvant chemoradiotherapy			
Performed	8 (80)	0 (0)	0.001
Not performed	2 (20)	10 (100)	
Adjuvant chemotherapy			
Performed	10 (100)	6 (60)	0.087
Not performed	0 (0)	4 (40)	
Time interval between *t*_*1*_ and *t*_*2*_ (months)	6.0 ± 1.9	8.0 ± 2.1	0.038

Data are presented as means ± standard deviations or numbers (percentages)

BMI, body mass index; ASA, American Society of Anesthesiologists; AR, anterior resection; LAR, low anterior resection; uLAR, ultra-low anterior resection; TNM, tumor-node-metastasis

### Alpha diversity analysis—*t*_1_ vs. *t*_2_

**Fig. 1:**
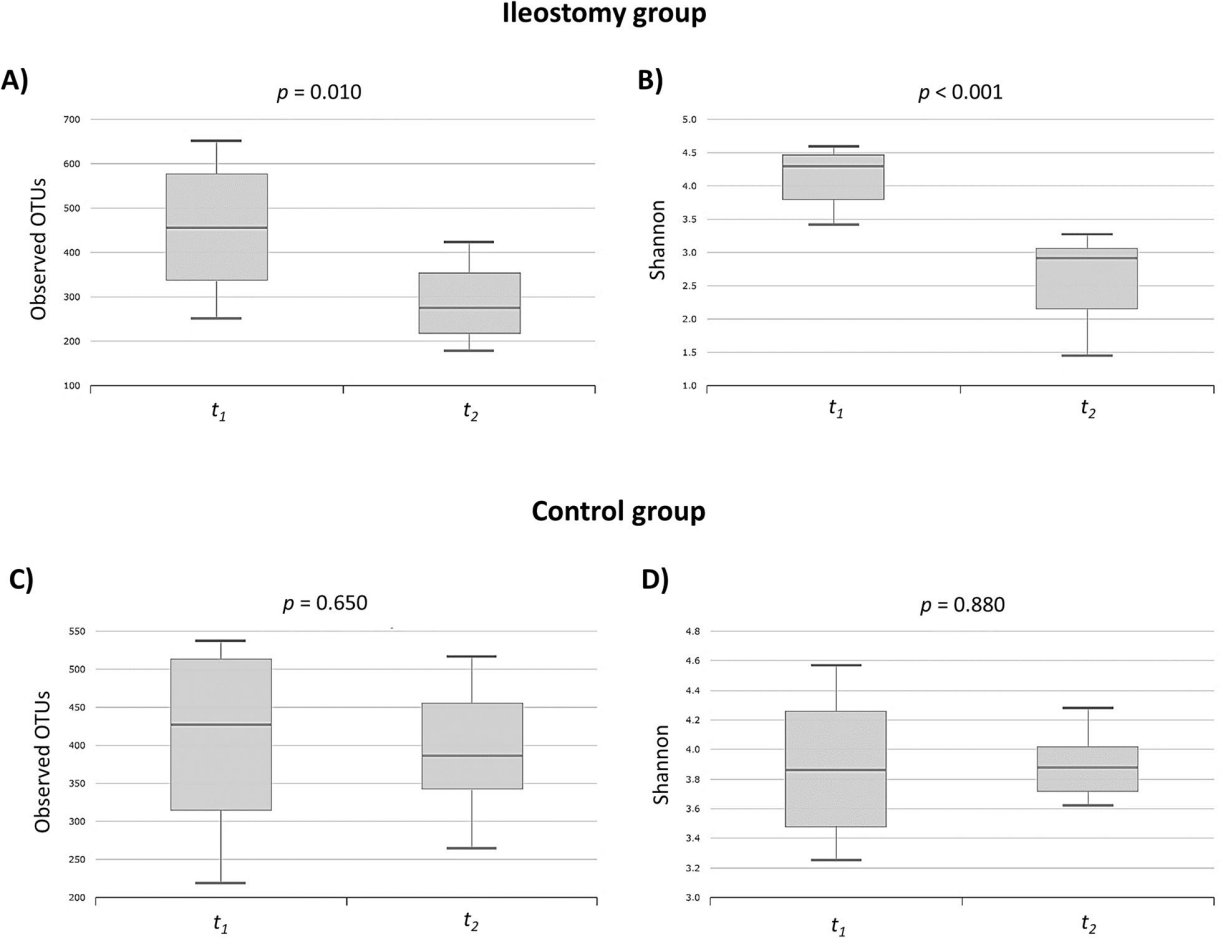
Alpha diversity analysis: comparison between *t*_*1*_ and *t*_*2*_ in the ileostomy (**A**, **B**) and control (**C**, **D**) groups. Within-sample diversities were measured by observed operational taxonomic units (OTUs) (**A**, **C**) and the Shannon index (**B**, **D**)

In the ileostomy group, the complexity within the samples significantly decreased after ileostomy repair (*t*_*2*_) compared with that before the initial surgery (*t*_*1*_) in terms of the observed operational taxonomic units (OTUs) (*p* = 0.010) and Shannon index (*p* < 0.001). However, in the control group, no significant differences were observed in the OTUs (*p* = 0.650) and Shannon index (*p* = 0.880) between *t*_*1*_ and *t*_*2*_ (Fig. 1).

### Alpha diversity analysis—ileostomy vs. control groups

Before the initial surgery (*t*_*1*_), the two groups showed no significant differences in the complexity within samples (OTUs, *p* = 0.406; Shannon index, *p* = 0.226). However, at *t*_*2*_, the ileostomy group had significantly lower complexity than the control group (OTUs, *p* = 0.010; Shannon index, *p* < 0.010) (Additional file 1: Fig. S1).

### Beta diversity analysis—*t*_1_ vs. *t*_2_

**Fig. 2:**
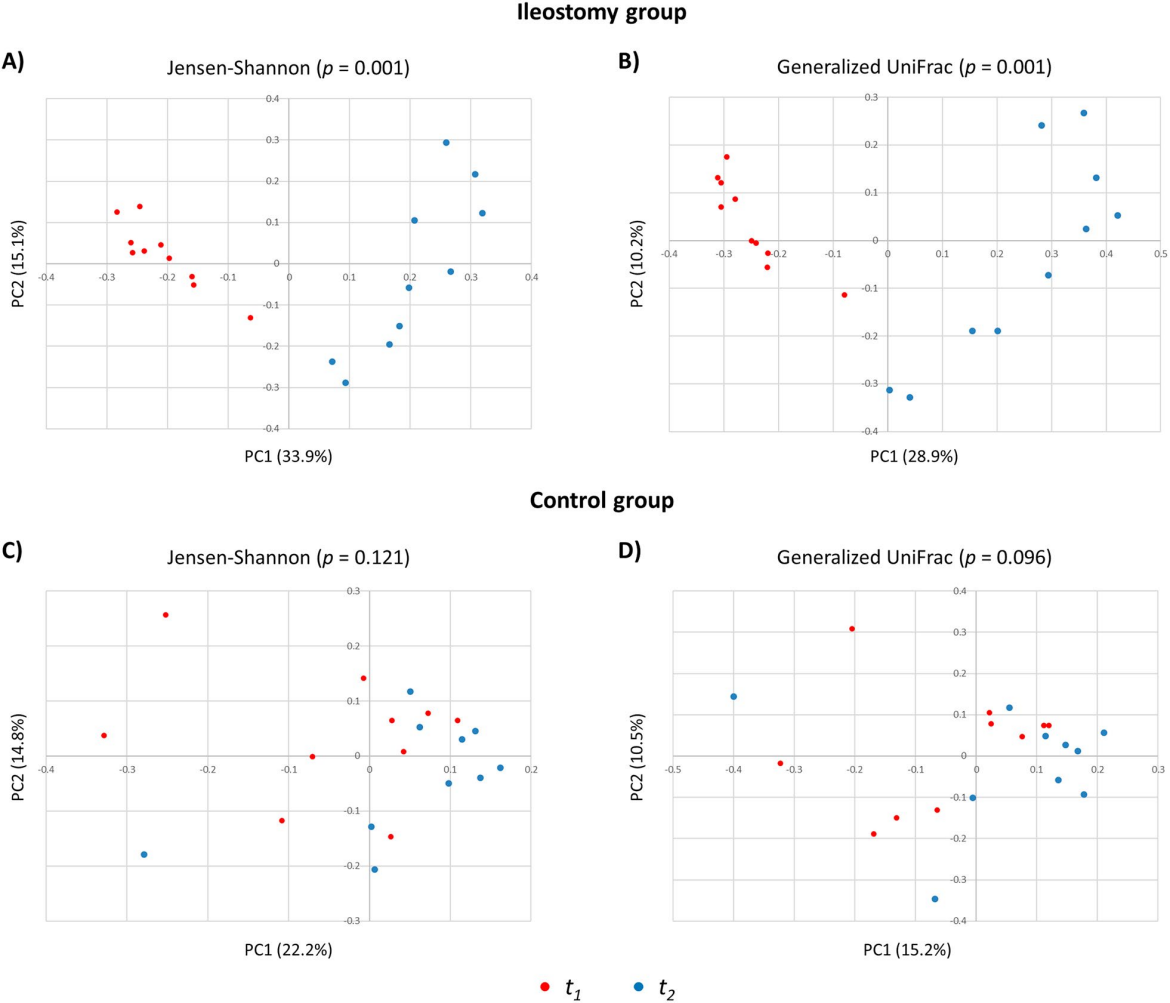
PCoA 2D plots of beta diversity analysis: comparison between *t*_*1*_ and *t*_*2*_ in the ileostomy (**A**, **B**) and control (**C**, **D**) groups. Between-sample dissimilarities were measured by the Jensen-Shannon divergence (**A**, **C**) and generalized UniFrac distance (**B**, **D**)

In the ileostomy group, principal coordinate analysis (PCoA) showed significant dissimilarities of the gut microbiota between the test before the initial surgery (*t*_*1*_) and after ileostomy repair (*t*_*2*_) (Jensen-Shannon, *p* = 0.001; generalized UniFrac, *p* = 0.001). However, in the control group, the beta diversity showed no significant dissimilarities between *t*_*1*_ and *t*_*2*_ (Jensen-Shannon, *p* = 0.121; generalized UniFrac, *p* = 0.096) (Fig. 2).

### Beta diversity analysis—ileostomy vs. control groups

At baseline (*t*_*1*_), the beta diversity showed no dissimilarities between the two groups (Jensen-Shannon, *p* = 0.501; generalized UniFrac, *p* = 0.470). However, at *t*_*2*_, the two groups had significant dissimilarities of the gut microbiota (Jensen-Shannon, *p* = 0.001; generalized UniFrac, *p* = 0.001) (Additional file 2: Fig. S2).

### Composition of the fecal microbiota—*t*_1_ vs. *t*_2_

**Fig. 3:**
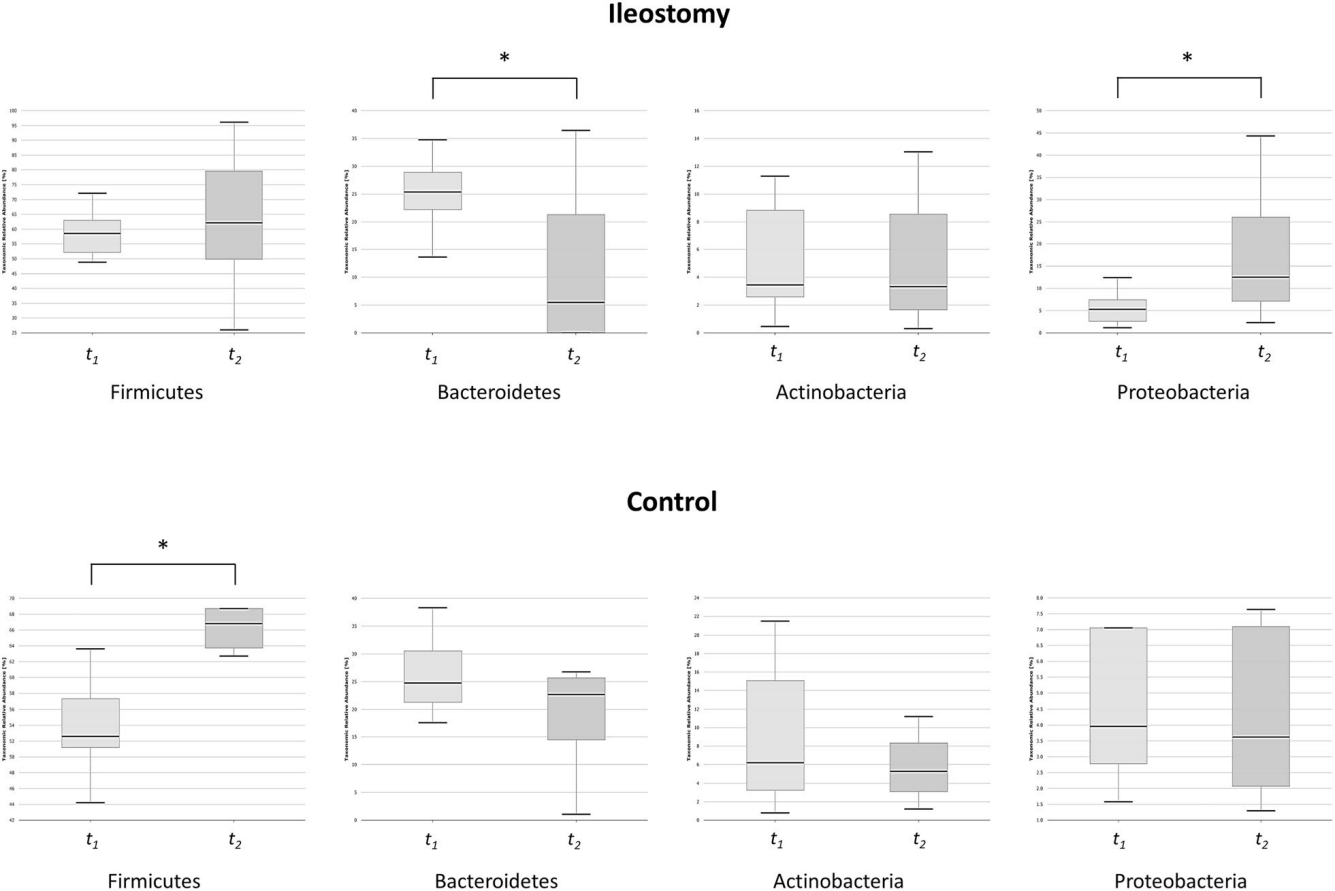
Relative abundance of bacteria at the phylum level: comparison between *t*_*1*_ and *t*_*2*_ in the ileostomy and control groups (*p < 0.05)

At the phylum level, in the ileostomy group, there was a significant decrease in the relative abundance of Bacteroidetes (26.1% vs. 12.1%, *p* = 0.023) and a significant increase in the relative abundance of Proteobacteria (5.8% vs. 17.9%, *p* = 0.016) between baseline (*t*_*1*_) and the time of ileostomy repair (*t*_*2*_) (Fig. 3). The Firmicutes/Bacteroidetes (F/B) ratio was significantly higher at *t*_*2*_ [median 21.6, interquartile range (IQR) 3.19–915.5] than at *t*_*1*_ (median 2.11, IQR 1.96–2.93, *p* = 0.034). However, in the control group, the relative abundance of Firmicutes significantly increased between *t*_*1*_ and *t*_*2*_ (54.4% vs. 67.5%, *p* = 0.010), while no significant difference was observed in the relative abundance of other phyla (Fig. 3).

At the family level, the changes in Lachnospiraceae and Ruminococcaceae were noticeable. Between *t*_*1*_ and *t*_*2*_, a significant decrease was observed in the proportions of Lachnospiraceae (29.7% vs. 3.8%, p < 0.001) and Ruminococcaceae (16.5% vs. 0.6%, p < 0.001) in the ileostomy group, whereas no significant difference was observed in the control group (Lachnospiraceae, 22.0% vs. 29.9%, *p* = 0.050; Ruminococcaceae, 12.6% vs. 18.4%, *p* = 0.082).

**Fig. 4:**
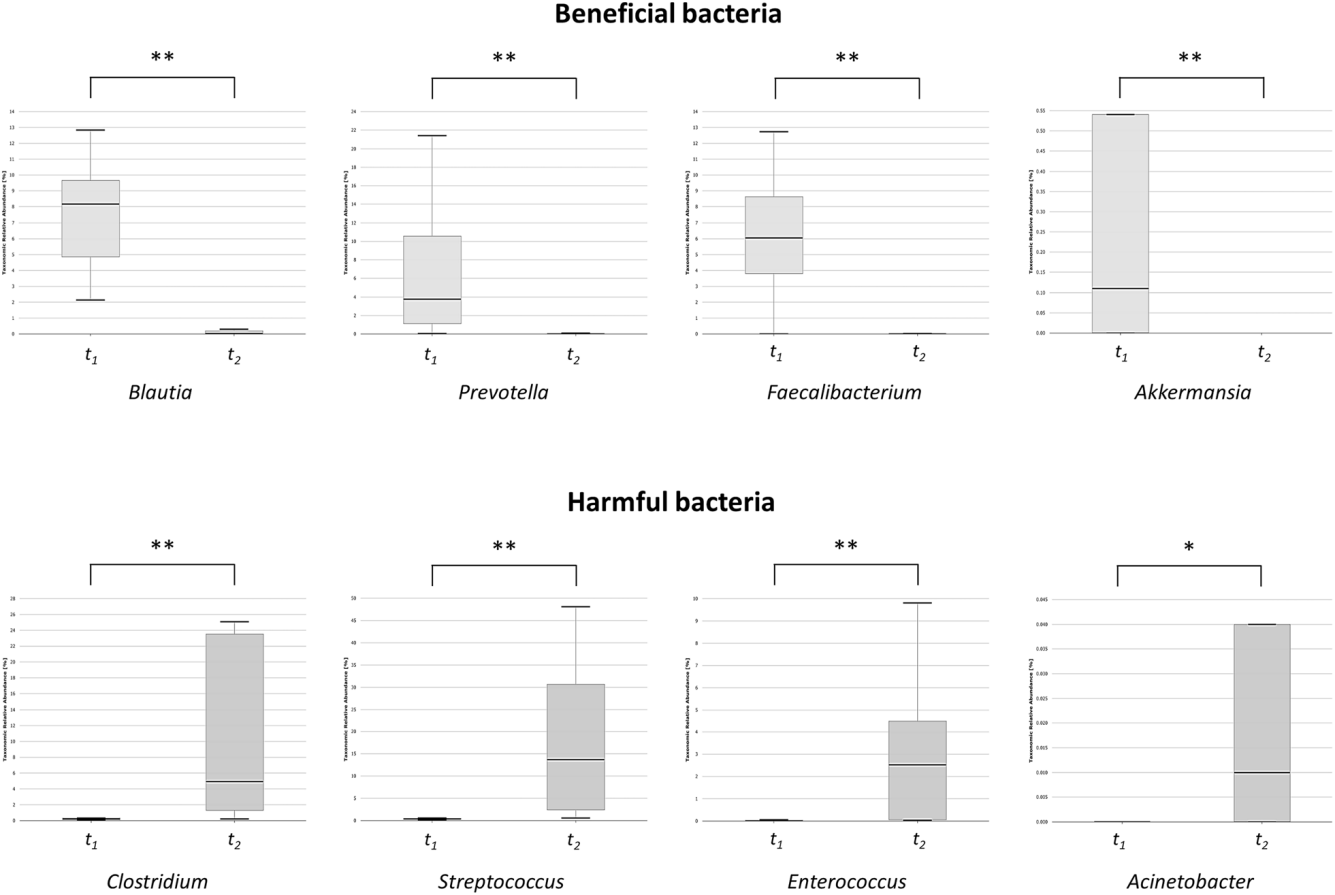
Relative abundance of bacteria at the genus level: comparison between *t*_*1*_ and *t*_*2*_ in the ileostomy group (*p < 0.05, **p < 0.01)

At the genus level, in the ileostomy group, the proportions of beneficial bacteria such as *Blautia* (7.4% vs. 0.1%, *p* < 0.001), *Prevotella* (6.8% vs. 0.0%, *p* = 0.001), *Faecalibacterium* (6.0% vs. 0.2%, *p* = 0.002), and *Akkermansia* (0.8% vs. 0.0%, *p* = 0.002) decreased, whereas those of harmful bacteria such as *Clostridium* (0.8% vs. 16.2%, *p* = 0.005), *Streptococcus* (1.1% vs. 17.7%, *p* = 0.001), *Enterococcus* (0.1% vs. 3.7%, *p* = 0.001), and *Acinetobacter* (0.0% vs. 3.3%, *p* = 0.044) increased while the ileostomy was maintained between *t*_*1*_ and *t*_*2*_ (Fig. 4). In contrast, no specific tendency was found in the control group. Some beneficial bacteria increased (*Faecalibacterium*, 3.8% vs. 7.5%, p = 0.019), while some other beneficial and harmful bacteria decreased (*Prevotella*, 7.4% vs. 2.6%, p = 0.028; *Streptococcus*, 4.2% vs. 1.6%, *p* = 0.019) (Additional file 3: Fig. S3).

### Composition of the fecal microbiota—ileostomy vs. control group

The composition of the fecal microbiota was generally similar between the two groups at *t*_*1*_. However, at *t*_*2*_, a significant difference was observed in the proportion of the Proteobacteria (17.9% vs. 5.1%, *p* = 0.006) phylum between the ileostomy and control groups (Additional file 4: Fig. S4). In addition, the F/B ratio was higher in the ileostomy group (median 21.6, IQR 3.19–915.5) than in the control group (median 2.75, IQR 2.48–5.39) at *t*_*2*_; however, this difference was not found to be statistically significant (*p* = 0.131).

At the family level, the ileostomy group had a lower proportion of beneficial bacteria such as Lachnospiraceae (3.8% vs. 29.9%, *p* < 0.001) and Ruminococcaceae (0.6% vs. 18.4%, *p* < 0.001) and a higher level of Streptococcaceae (18.7% vs. 1.7%, *p* = 0.002) and Clostridiaceae (16.2% vs. 1.1%, *p* = 0.013) than the control group at *t*_*2*_.

**Fig. 5:**
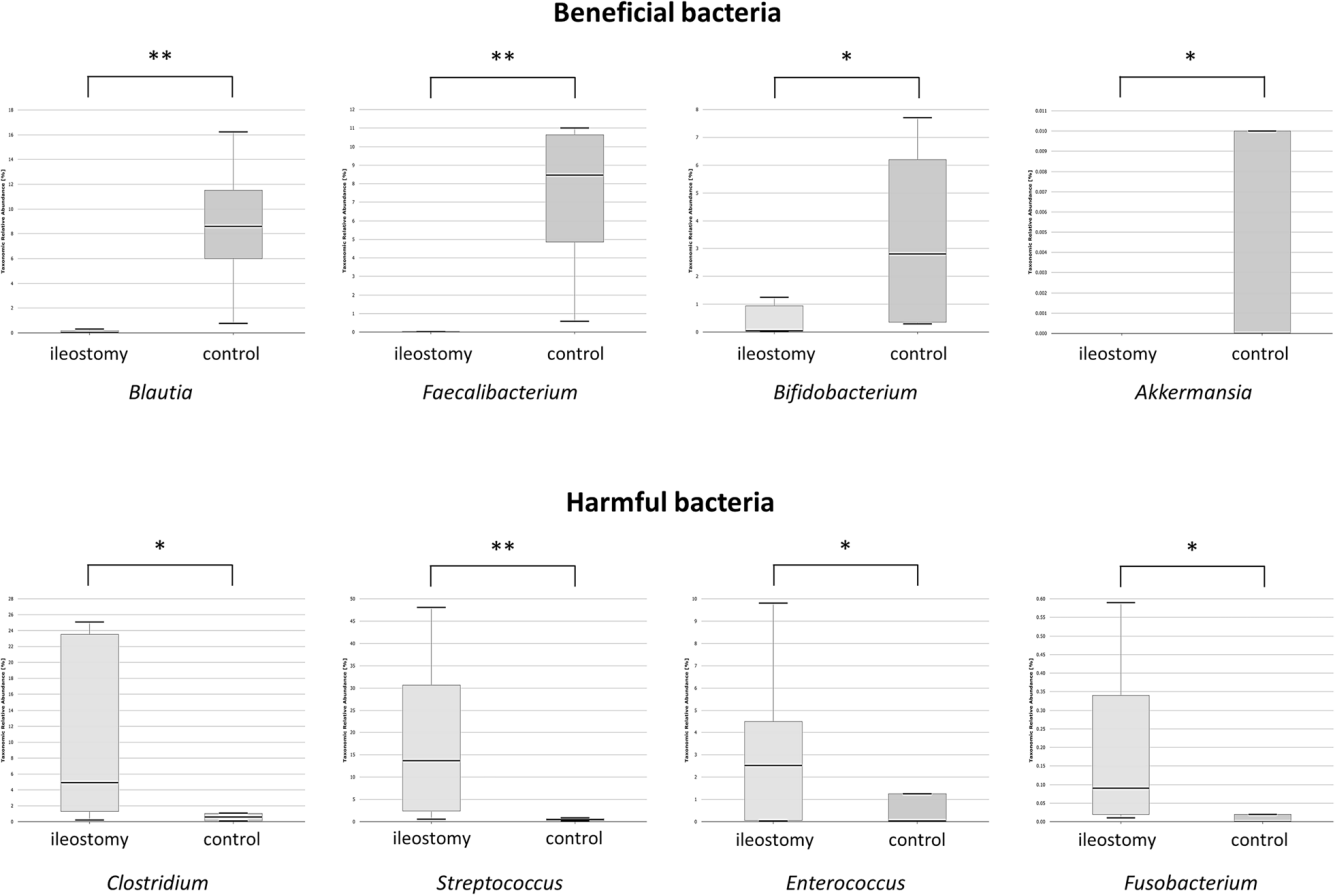
Relative abundance of bacteria at the genus level: comparison between the ileostomy and control groups, at *t*_*2*_ (*p < 0.05, **p < 0.01)

At the genus level, the proportions of beneficial bacteria such as *Blautia* (0.1% vs. 9.1%, *p* < 0.001), *Faecalibacterium* (0.2% vs. 7.5%, p < 0.001), *Bifidobacterium* (0.6% vs. 4.8%, *p* = 0.01), and *Akkermansia* (0.0% vs. 0.1%, *p* = 0.013) were significantly lower, while those of harmful bacteria such as *Clostridium* (16.2% vs. 1.1%, *p* = 0.013), *Streptococcus* (17.7% vs. 1.6%, *p* = 0.002), *Enterococcus* (3.7% vs. 1.5%, *p* = 0.049), and *Fusobacterium* (1.5% vs. 0.3%, *p* = 0.019) were higher in the ileostomy group than those in the control group at *t*_*2*_ (Fig. 5).

**Fig. 6:**
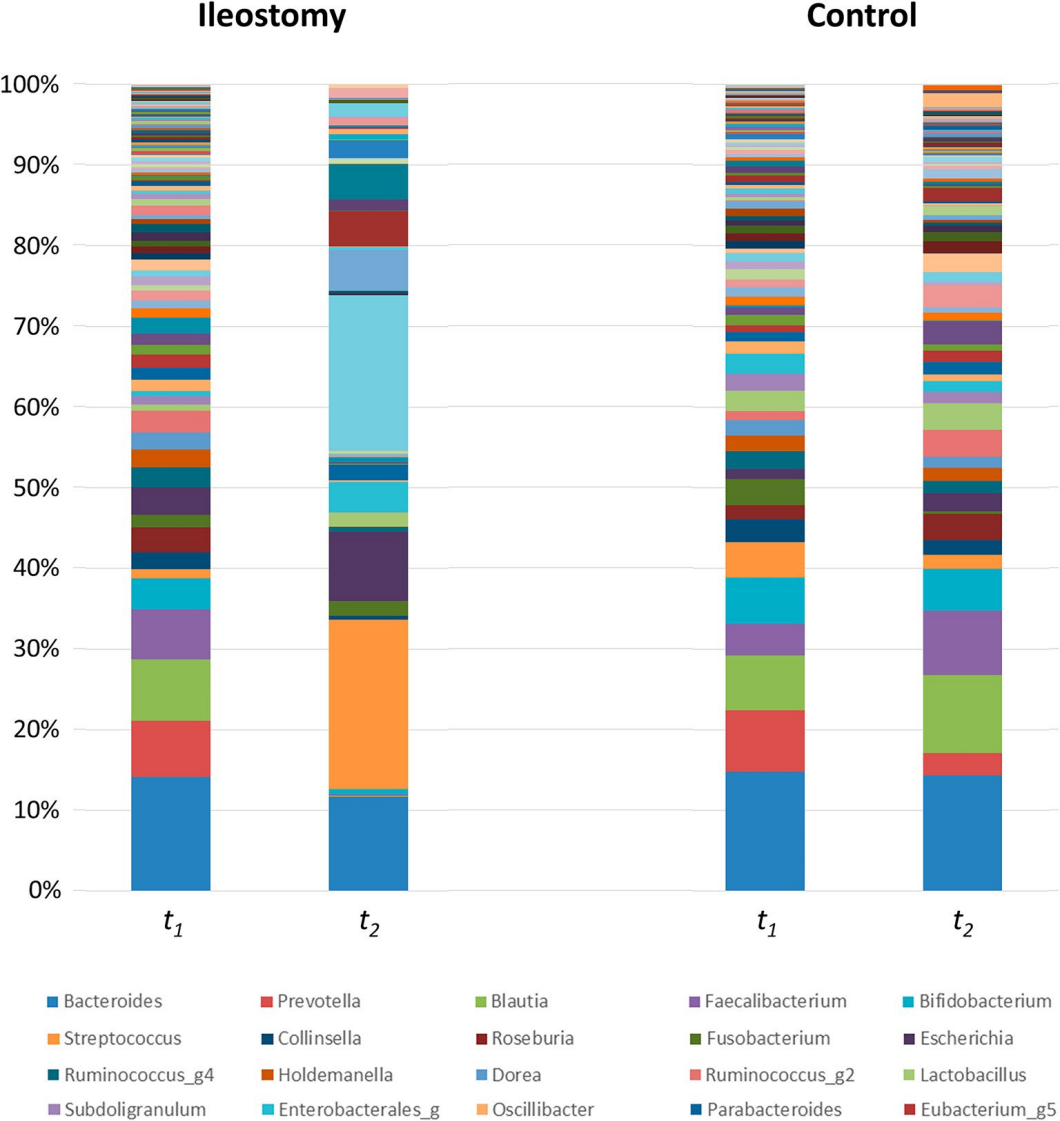
Composition of the bacterial community at the genus level for the ileostomy and control groups at *t*_*1*_ and *t*_*2*_. The legend on the inferior side represents the 20 most abundant genera arranged in order of frequency

The compositions of the bacterial community at the level of genus, phylum, and species for the control and ileostomy groups at *t*_*1*_ and *t*_*2*_ are depicted in Fig. 6 and Additional file 5: Fig. S5 and Additional file 6: Fig. S6.

## Discussion

The present study investigated the effect of fecal diversion on the gut microbiota in patients with colorectal cancer. The results showed that patients with a diverting stoma had a reduction in gut microbiota diversity, a decrease in beneficial bacteria, and an increase in harmful bacteria, leading to dysbiosis of the gut microbiota.

Although there have been limited investigations regarding the impact of fecal stream diversion on the gut microbiota, all existing studies have reported microbiota associated with tissue in the ileum [11, 13]. Furthermore, the loop ileostomy creates an environment where the colon, which houses a vast collection of commensal microbiota producing fermentation substances including short-chain fatty acids, bile acids, and tryptophan, is devoid of a fecal stream [12]. The intestinal commensal microbiota and their fermentation products are crucial in maintaining intestinal homeostasis and integrity; therefore, further exploration of the effect of fecal stream diversion on the fecal microbiota is warranted. In the present study, the alpha diversity of the fecal microbiota was observed to decrease during the maintenance of a diverting stoma, in accordance with the findings of previous studies that reported decreased diversity of the tissue-associated microbiota [11, 13]. In addition, we also found a change in the microbial community structure in the ileostomy group as assessed by the beta diversity. Collectively, our results confirmed that fecal stream diversion led to dysbiosis of the gut microbiota, as evidenced by fecal testing.

A comprehensive investigation of alterations of the gut microbiota can provide a more nuanced understanding of the progression of dysbiosis while maintaining a diverting stoma. At the phylum level, the proportion of Bacteroidetes decreased, while that of Proteobacteria increased up to 17.9% during fecal stream diversion in the ileostomy group. The increased abundance of Proteobacteria has commonly been linked to various conditions, including obesity, metabolic disorders, inflammation, and cancer [14]. It has been observed that undernourished children tend to exhibit an overrepresentation of Proteobacteria and low diversity in their gut microbiota [15, 16], suggesting that an abundance of gut Proteobacteria is reflective of both an energy imbalance in the host and an unstable microbial community [14]. Although these bacteria are typically harmless when present in small proportions, under specific gut conditions, they can transform into colitogenic microbes capable of provoking inflammatory reactions [14]. Furthermore, the F/B ratio was significantly higher at *t*_*2*_ than at *t*_*1*_. The phyla Firmicutes and Bacteroidetes are the most dominant bacteria in the gut microbiota, accounting for 90% of its composition [17]. The F/B ratio has been known to be associated with maintaining homeostasis within the gut microbiota [17, 18]. In metabolic disorders, dysbiosis of the gut microbiota is frequently marked by an elevated F/B ratio, suggesting that imbalances in the populations of Firmicutes and Bacteroidetes could play a crucial role in the pathophysiology of these conditions [18, 19].

At the family level, the decrease in the proportions of normal commensal bacterial microbiota such as Lachnospiraceae and Ruminococcaceae between *t*_*1*_ and *t*_*2*_ was notable in the ileostomy group. Lachnospiraceae and Ruminococcaceae are prominent bacterial families known for their ability to ferment complex polysaccharides into simpler compounds such as short-chain fatty acids, which can be used by the host as an energy source [20]. The observation of alterations in the abundance and diversity of Lachnospiraceae across various diseases, such as inflammatory bowel disease, obesity, and diabetes, highlights the vital role of this family in preserving gut health and mitigating disease development [20]. The present study also demonstrated that the maintenance of fecal diversion resulted in an increase in the proportions of some genera, such as *Clostridium* and *Streptococcus*, and a decrease in others, such as *Blautia*, *Prevotella*, and *Faecalibacterium*. While certain species of *Clostridium* are beneficial for gut health, others, such as *C. difficile* and *C. perfringens*, can have negative impacts and lead to infections [21]. *Blautia*, a prominent genus of the gut microbiota, has been found to have positive effects on host health, including the ability to regulate metabolic syndrome and biotransformation [22]. The abundance of *Prevotella* in the healthy gut microbiota as well as its association with plant-rich diets have led to its classification as a potentially beneficial genus of bacteria [23]. *Faecalibacterium prausnitzii*, one of the most important butyrate-producing bacteria in the human colon, has been identified as an indicator of human health [24]. Overall, fecal stream diversion resulted in a shift towards dysbiosis of the gut microbiota characterized by an increase in harmful bacteria and a decrease in beneficial bacteria.

Dysbiosis of the gut microbiota can be associated with various symptoms or postoperative complications regarding maintenance and reversal of a diverting ileostomy. Given the significant alteration in the gut microbiota observed in the defunctioned colon in the present study, it is reasonable to assume that changes in the gut microbiota will have a substantial impact on the development of diversion colitis. In addition, changes in the gut microbiota may have an impact on the occurrence of postoperative complications following ileostomy reversal. Numerous studies have reported that the composition of the gut microbiota could play a crucial role in determining the outcomes of gastrointestinal surgery [25, 26]. As the human microbiota constitutes a significant element of the host's immune system, preserving the fundamental structure of the gut microbiota is critical in preventing severe infections [25]. As a representative example, the composition of the gut microbiota has a significant impact on the development of *C. difficile* infection [27], which is known to occur more frequently following ileostomy reversal surgery [28]. In addition, alterations in the composition of the gut microbiota, along with luminal shrinkage and impaired motility, may contribute to the development of postoperative ileus following ileostomy reversal [29].

Researchers have attempted several approaches to mitigate these complications following ileostomy reversal, including the stimulation of the defunctioned bowel using saline or diluted ileostomy output [29] and preoperative administration of probiotics [30, 31]. However, these interventions currently lack robust clinical evidence. Yoon et al. investigated the efficacy of probiotics in restoring bowel function after ileostomy closure; however, their findings did not reveal significant effects supporting the use of probiotics for improved bowel function [30]. Moreover, studies on the use of probiotics in colorectal cancer surgery have shown inconsistent results [32, 33]. The results of our study can serve as a theoretical foundation for setting the direction of future research. In addition, we are planning a follow-up study to investigate the changes in fecal microbiota when probiotics are administered during postoperative period after ileostomy closure.

The limitations of the present study are evident in the small sample size of 20 participants, which raises concerns about the generalizability of the findings. Nevertheless, the results of the study notably exhibited a significant effect size, suggesting that the sample size was sufficient to draw meaningful conclusions. Further examination on a larger number of patients in the future will verify and strengthen the findings of this study. Furthermore, the presence of notable variations in baseline characteristics, including nCRT and the time interval between *t*_*1*_ and *t*_*2*_, could have potentially influenced the outcome of the study. To account for these potential confounding factors, a comparative analysis that considered the differences in baseline characteristics and a time series comparison of the outcomes were conducted between the ileostomy and control groups. This comprehensive analysis revealed that the observed alteration in the gut microbiota of the ileostomy group was not attributable to baseline characteristic disparities but rather to the effect of fecal diversion itself.

## Conclusion

Fecal stream diversion was found to be significantly associated with reduced diversity and dysbiosis of the gut microbiota. The comprehensive insight of our study into the effect of fecal stream diversion on the gut microbiota has significant implications for managing dietary interventions in patients with colorectal cancer and other patient groups, such as those with eating disorders, with potential directions for future research in dietary interventions and gut health.

## Methods

### Participants and study design

**Fig. 7:**
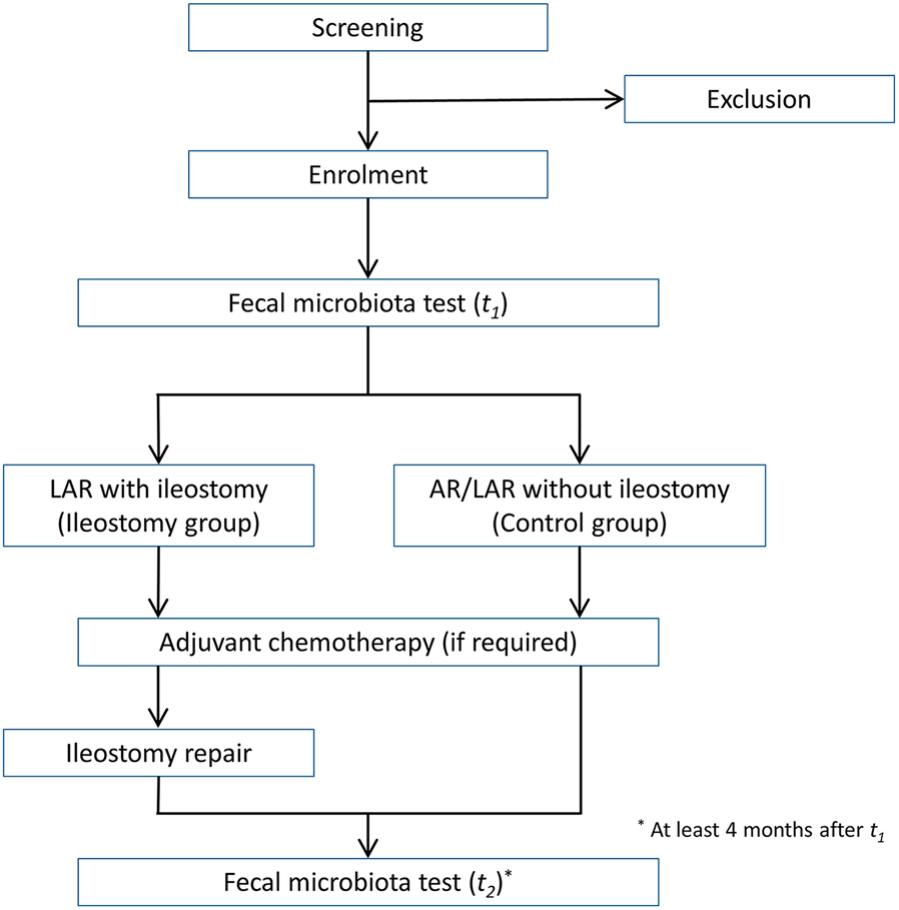
Study design

The present study was designed as a prospective cohort study and was approved by the institutional review board of our institution (IRB no. CNUHH-2019-215). We enrolled 20 consecutive patients who were scheduled to undergo left-sided colorectal cancer surgery. The inclusion criteria were patients aged 20–80 years with primary colorectal cancer. Those who were pregnant, who underwent emergency surgery, who had undergone previous stoma surgery, or who were scheduled to undergo permanent stoma surgery were excluded. The enrolled patients were divided into two groups (10 patients in each group): those undergoing surgery with diverting ileostomy (ileostomy group) and without ileostomy (control group) (Fig. 7). All the enrolled patients were informed about the study protocol and signed an informed consent form.

### Preoperative evaluation and neoadjuvant therapy

Abdominopelvic and chest computed tomography, colonoscopy, and laboratory tests including measurement of serum levels of carcinoembryonic antigen were performed on all the included patients. Pelvic magnetic resonance imaging was also performed on patients with rectal cancer. For patients with locally advanced mid-to-low rectal cancer (≥ cT3 or ≥ cN1), neoadjuvant chemoradiation (nCRT) (5040 cGy + capecitabine) was administered, and radical surgery was performed 6–10 weeks after the completion of nCRT.

### Surgical intervention and perioperative care

Mechanical bowel preparation using polyethylene glycol was performed the day before surgery. As a prophylactic antibiotic treatment, a second-generation cephalosporin was administered immediately before surgery. All surgical interventions were performed by a single colorectal surgical specialist according to oncological principles via a laparoscopic approach. Colorectal or coloanal anastomosis was performed using a double-stapling or hand-sewing method in the case of very low-lying rectal cancer. Diverting ileostomy was planned at the discretion of the attending surgeon for patients who underwent nCRT or those with advanced upper rectal cancer. Oral intake was started the day after surgery if obstructive symptoms were not reported.

For patients with locally advanced rectal cancer or those who received nCRT, 5-fluorouracil-based adjuvant chemotherapy was administered, according to the current National Comprehensive Cancer Network guidelines [34]. Ileostomy repair surgery was performed 4–10 months after the creation of diverting ileostomy and after the completion of adjuvant chemotherapy.

### Assessment

For microbiota testing, 20 fecal samples were collected preoperatively (*t*_*1*_) for all patients. For 10 patients in the ileostomy group, fecal samples were collected again immediately after ileostomy repair surgery (*t*_*2*_); in contrast, for the 10 other patients in the control group, fecal samples were collected 6–12 months after initial surgery (*t*_*2*_). Follow-up fecal samples (*t*_*2*_) were collected at least four months after initial surgery (*t*_*1*_) (Fig. 7). Changes in the composition of the gut microbiota were compared between the two groups.

### Fecal microbiota testing

We utilized QIAamp DNA Stool MiniKit (Qiagen®, Hilden, Germany) to extract genomic DNA from bacteria in the feces, according to the manufacturer’s instructions. After centrifuging, the library preparation was performed using polymerase chain reaction following the 16S Metagenomic Sequencing Library Preparation Illumina Protocol. The quality of the final library was assessed, and the amount of DNA was quantified using an Agilent Bioanalyzer 1000 (Agilent) and Qubit (Thermo Fisher Scientific Inc.). The detailed methods for fecal microbiota testing have been previously described [35]. The metagenome was analyzed using EzBioCloud (ChunLab, Inc.) and BaseSpace (Illumina) platform. Differences in the within-sample richness and evenness (alpha diversity) were analyzed using the Shannon index, and dissimilarities between samples (beta diversity) were analyzed using the Jensen-Shannon divergence and the generalized UniFrac distance.

### Statistical analysis

Categorical variables were compared using the χ^2^ test or Fisher’s exact test, and continuous variables were compared using Student’s *t*-test or the Wilcoxon rank sum test. The Kruskal–Wallis test and permutational multivariate analysis of variance (PERMANOVA) was performed to analyze the statistical significance of the alpha and beta diversities. All results were considered to be significant at a *p*-value of < 0.05. Statistical analyses were performed using SPSS version 27.0 (IBM Inc., Armonk, NY, USA).

### Supplementary Information

**Additional file 1: Fig. S1.** Alpha diversity analysis: comparison between the ileostomy and control groups at *t*_*1*_ (**A, B**) and *t*_*2*_ (**C, D**). Within-sample diversities were measured by observed operational taxonomic units (OTUs) (**A, C**) and the Shannon index (**B, D**).

**Additional file 2: Fig. S2.** PCoA 2D plots of beta diversity analysis: comparison between the ileostomy and control groups at *t*_*1*_ (**A, B**) and *t*_*2*_ (**C, D**). Between-sample dissimilarities were measured by the Jensen-Shannon divergence (**A, C**) and generalized UniFrac distance (**B, D**).

**Additional file 3: Fig. S3.** Relative abundance of bacteria at the genus level: comparison between *t*_*1*_ and *t*_*2*_ in the control group (*: *p* < 0.05).

**Additional file 4: Fig. S4.** Relative abundance of bacteria at the phylum level: comparison between the ileostomy and control groups at *t*_*1*_ and *t*_*2*_ (**: *p* < 0.01).

**Additional file 5: Fig. S5.** Composition of the bacterial community at the phylum level for the ileostomy and control groups at *t*_*1*_ and *t*_*2*_. The legend on the inferior side represents the 5 most abundant phyla arranged in order of frequency.

**Additional file 6: Fig. S6.** Stacked bar graphs of the relative abundance of bacteria at the species level for the ileostomy and control groups at *t*_*1*_ and *t*_*2*_. The legend on the inferior side represents the 20 most abundant species arranged in order of frequency.

## Data Availability

The DNA sequence data (fastq files) produced as part of this study are uploaded in NCBI SRA under Accession Number PRJNA948146.
