# Supplementary figures and images for: Dysbiosis of gut microbiota during fecal stream diversion in patients with colorectal cancer
Source: Gut Pathog. 2023 Aug 18;15:40. doi: 10.1186/s13099-023-00566-9 (PMC10439566; doi:10.1186/s13099-023-00566-9)

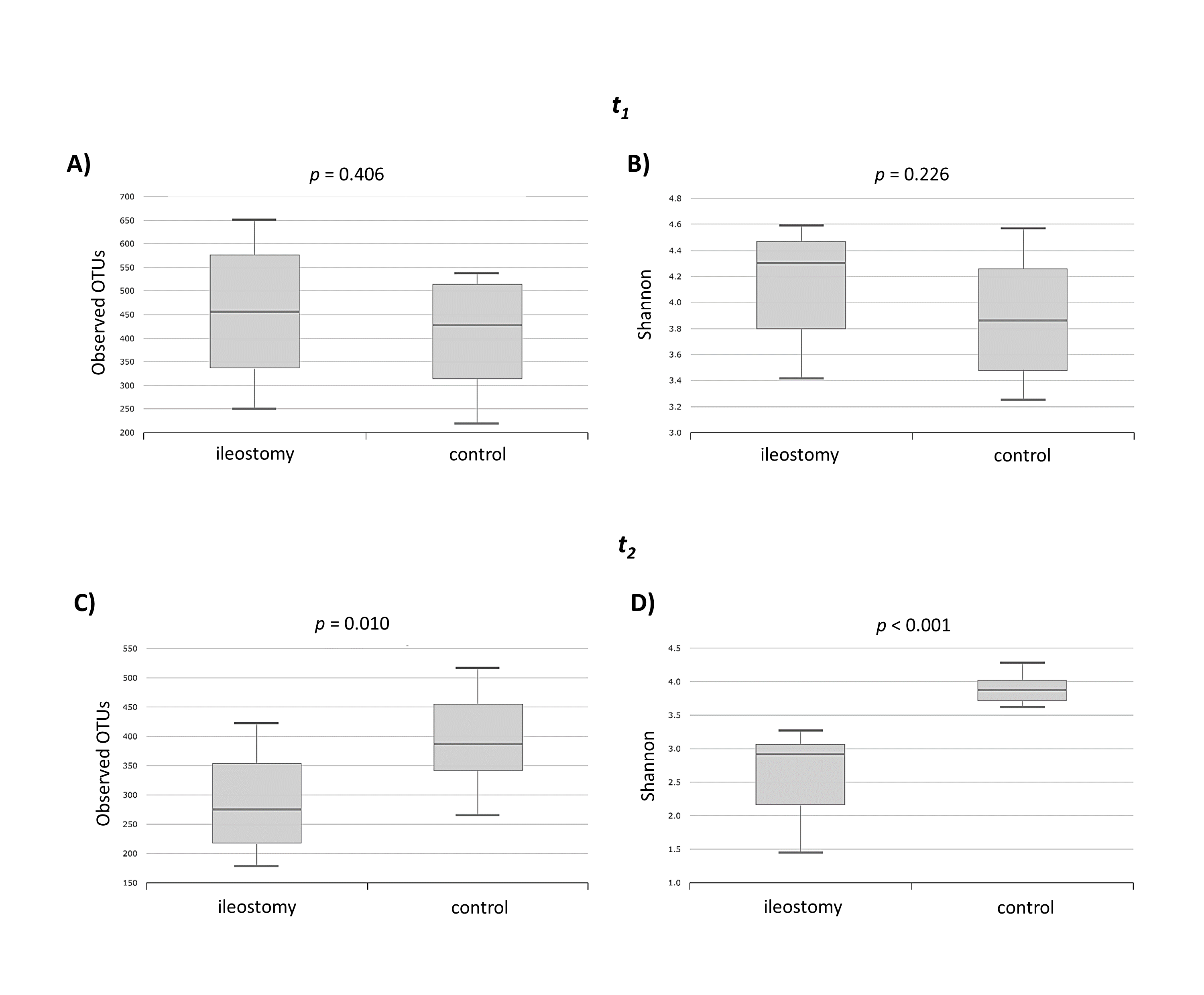

Supplement: Supplementary file 1 — Additional file 1: Fig. S1. Alpha diversity analysis: comparison between the ileostomy and control groups at t1 (A, B) and t2 (C, D). Within-sample diversities were measured by observed operational taxonomic units (OTUs) (A, C) and the Shannon index (B, D). [file 13099_2023_566_MOESM1_ESM.tif]

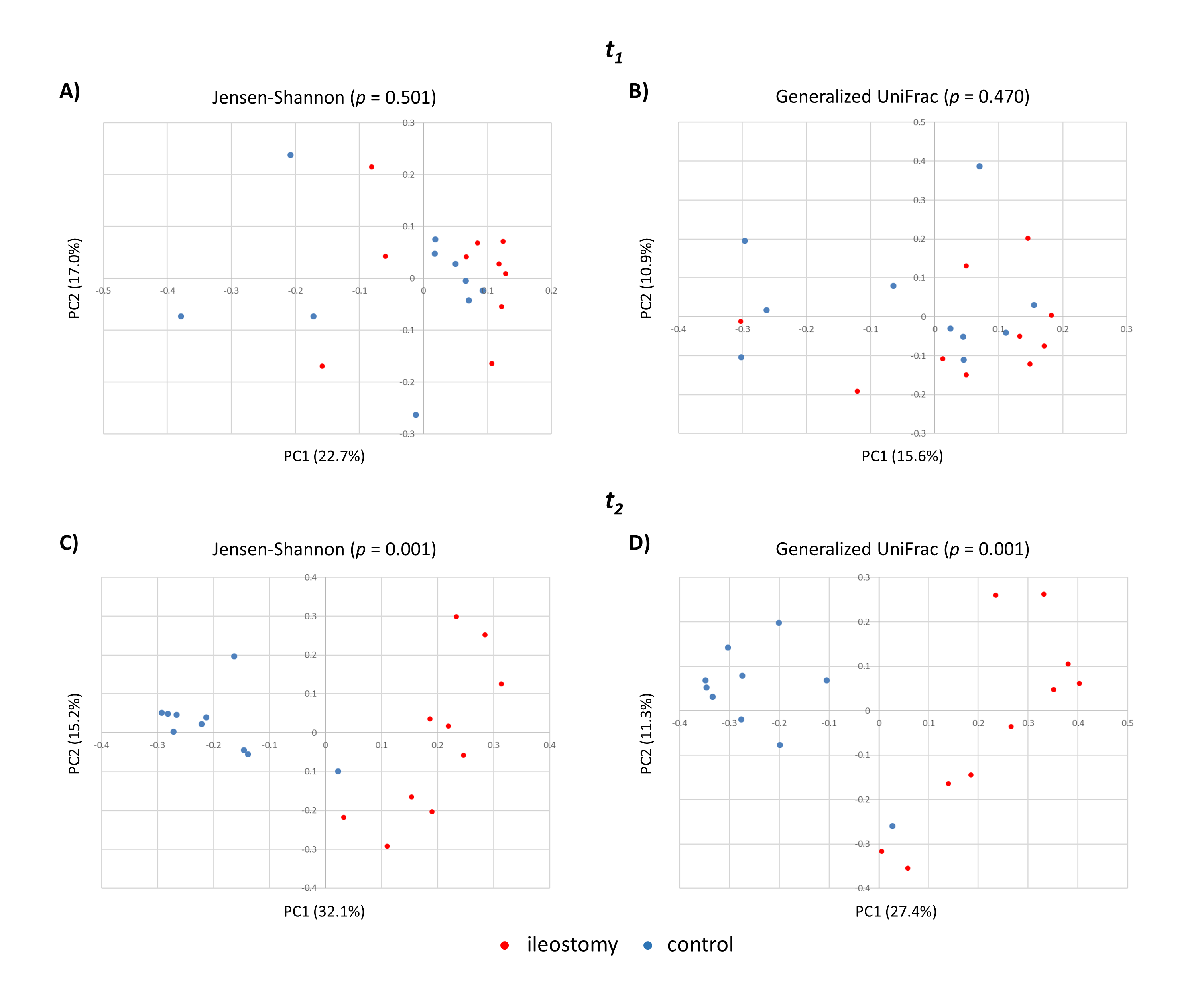

Supplement: Supplementary file 2 — Additional file 2: Fig. S2. PCoA 2D plots of beta diversity analysis: comparison between the ileostomy and control groups at t1 (A, B) and t2 (C, D). Between-sample dissimilarities were measured by the Jensen-Shannon divergence (A, C) and generalized UniFrac distance (B, D). [file 13099_2023_566_MOESM2_ESM.tif]

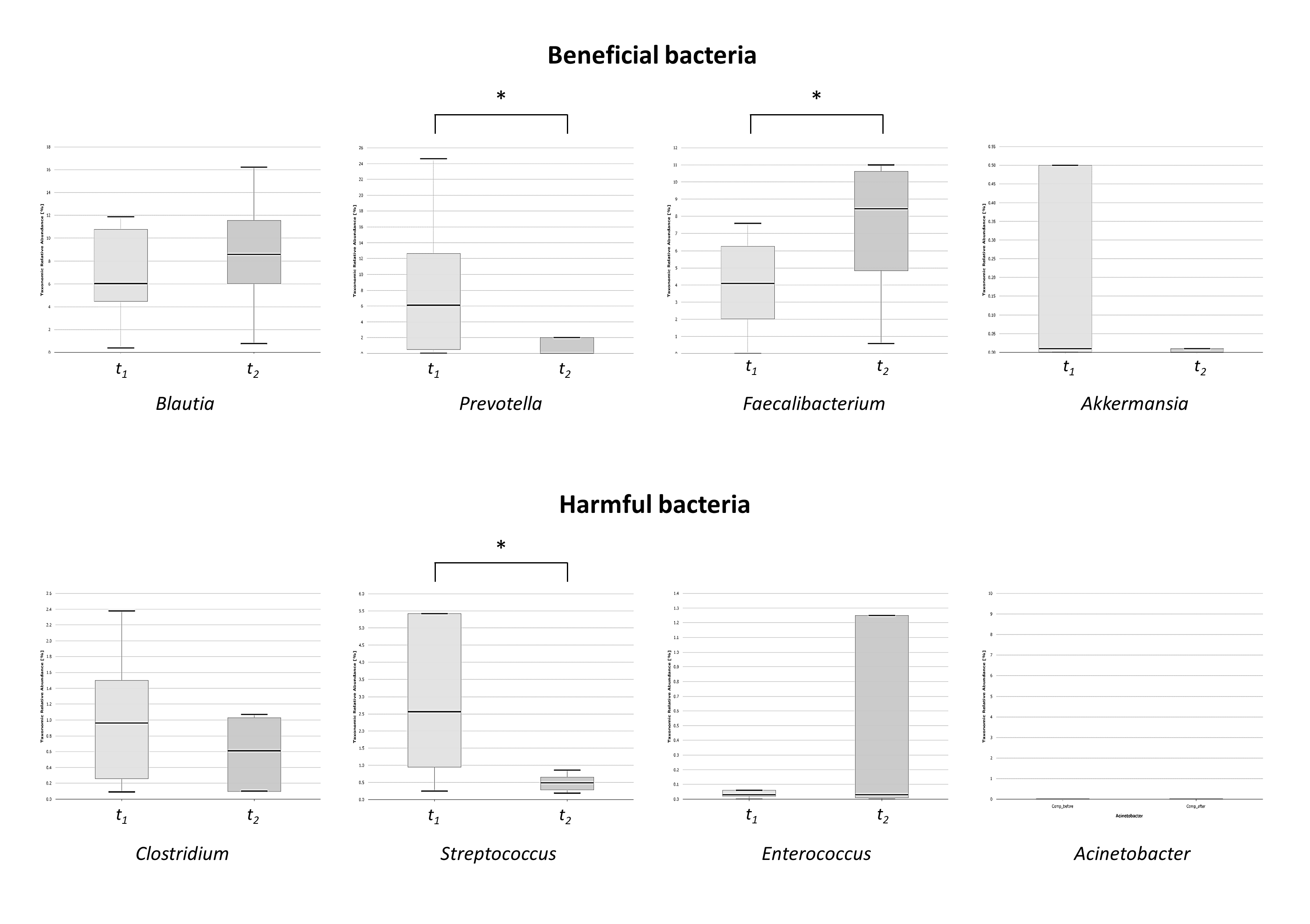

Supplement: Supplementary file 3 — Additional file 3: Fig. S3. Relative abundance of bacteria at the genus level: comparison between t1 and t2 in the control group (*: p < 0.05). [file 13099_2023_566_MOESM3_ESM.tif]

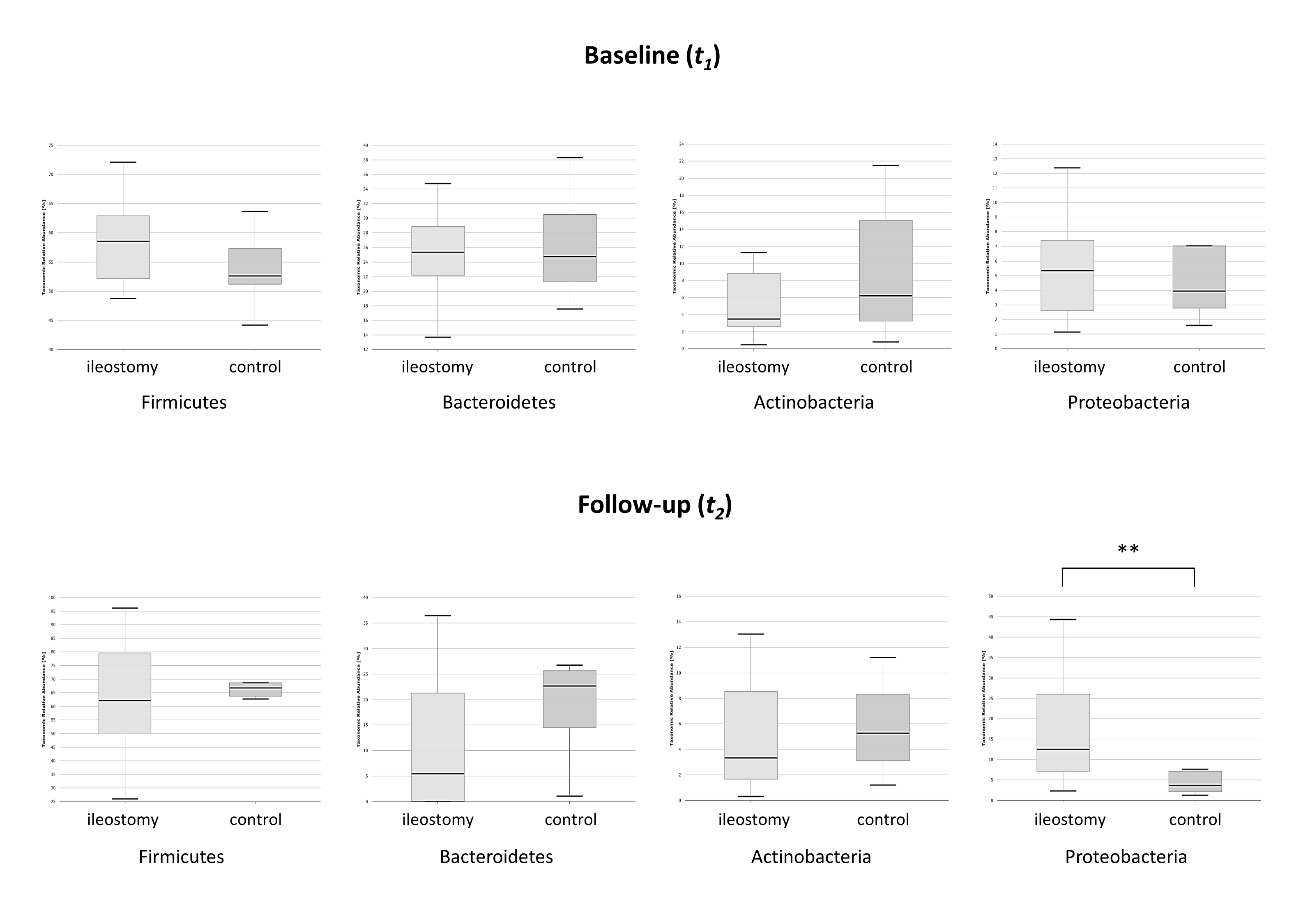

Supplement: Supplementary file 4 — Additional file 4: Fig. S4. Relative abundance of bacteria at the phylum level: comparison between the ileostomy and control groups at t1 and t2 (**: p < 0.01). [file 13099_2023_566_MOESM4_ESM.tif]

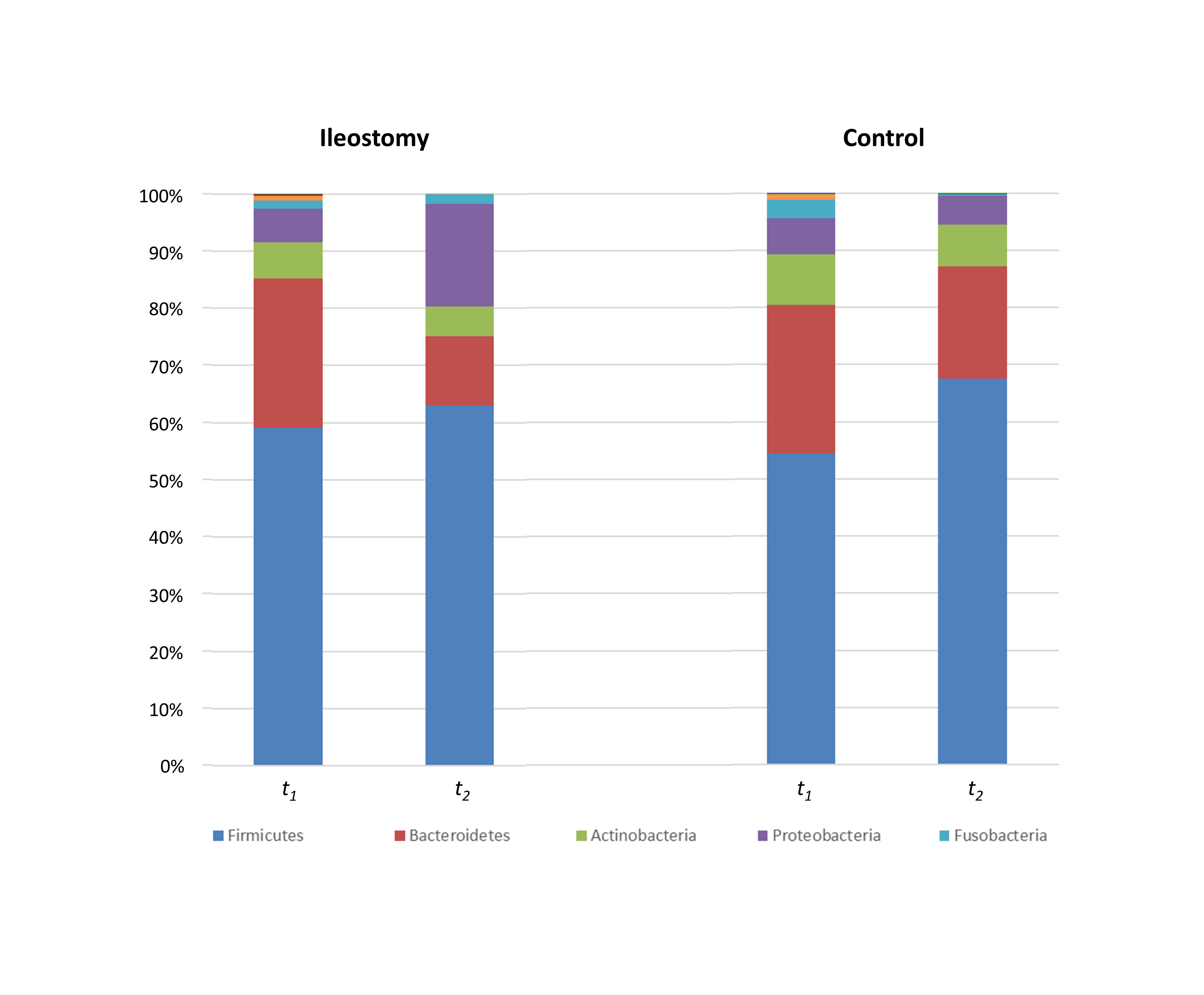

Supplement: Supplementary file 5 — Additional file 5: Fig. S5. Composition of the bacterial community at the phylum level for the ileostomy and control groups at t1 and t2. The legend on the inferior side represents the 5 most abundant phyla arranged in order of frequency. [file 13099_2023_566_MOESM5_ESM.tif]

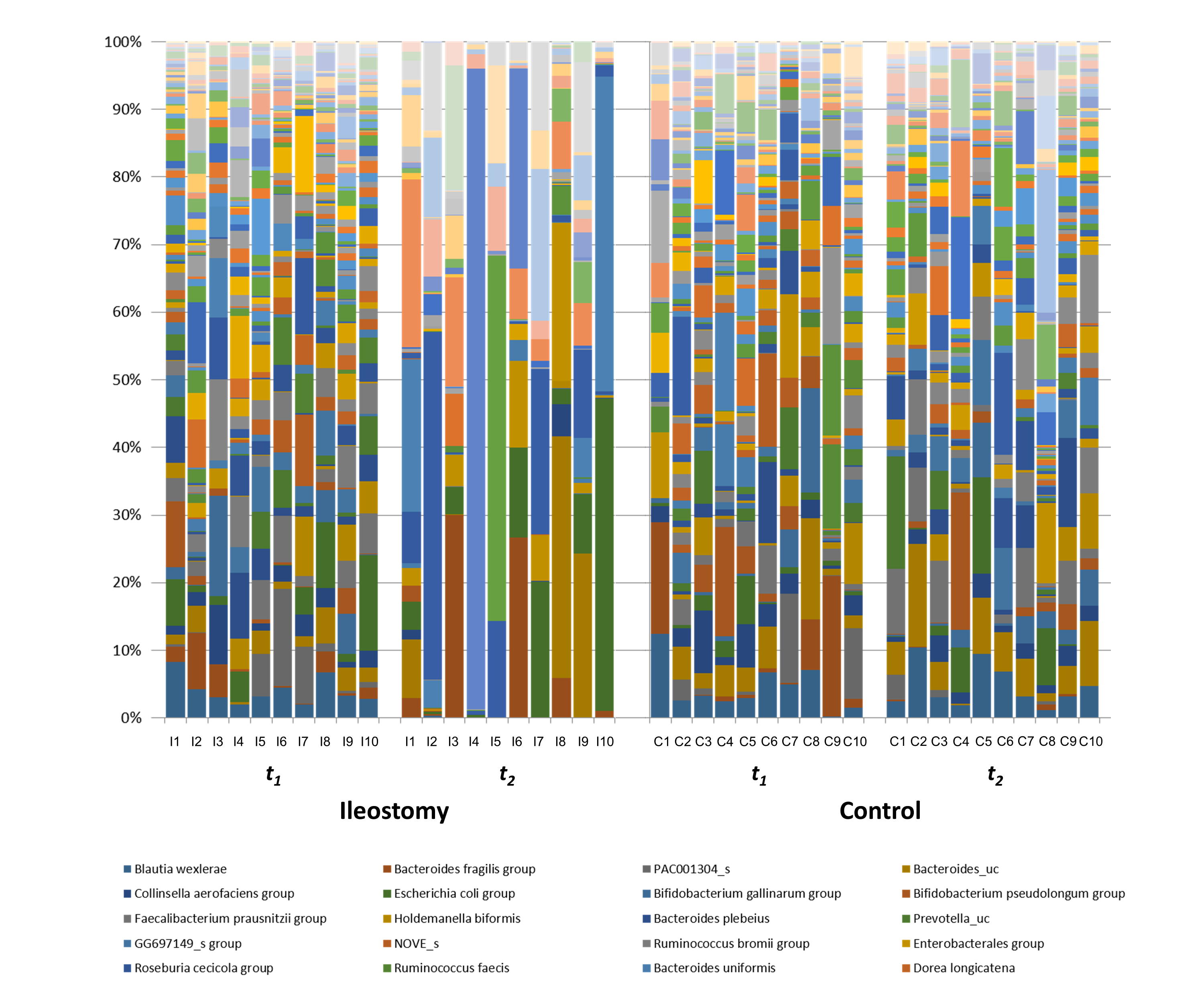

Supplement: Supplementary file 6 — Additional file 6: Fig. S6. Stacked bar graphs of the relative abundance of bacteria at the species level for the ileostomy and control groups at t1 and t2. The legend on the inferior side represents the 20 most abundant species arranged in order of frequency. [file 13099_2023_566_MOESM6_ESM.tif]
